# A Mini Review of Advances in Porous Materials Designing for Hydrogen Isotope Separation

**DOI:** 10.3390/ma17235708

**Published:** 2024-11-22

**Authors:** Huafeng Zhu, Liangbo Xu, Jia Li, Duanwei He, Jingchuan Wang

**Affiliations:** 1 Institute of Atomic and Molecular Physics, Sichuan University, Chengdu 201800, China; zhuhuaf@foxmail.com; 2 Institute of Materials Research, Chinese Academy of Engineering Physics, Jiangyou 621908, China; xuliangbo@stu.scu.edu.cn (L.X.); a15093270779@163.com (J.L.)

**Keywords:** hydrogen isotope separation (HIS), quantum sieving (QS), MOFs, zeolites

## Abstract

The separation of mixtures of hydrogen isotopes is one of the greatest challenges of modern separation technology. A newly proposed separation mechanism, the quantum sieving (QS) effect, is expected to achieve high separation factors, the main desired properties for hydrogen isotope separation (HIS). Metal–organic frameworks (MOFs) and zeolites are excellent candidates to study these quantum effects because of their well-defined and tunable pore structure and the potential to introduce strong adsorption sites directly into the framework structure. This paper briefly discusses the fundamentals of QS of hydrogen isotopes in nanoporous materials, mainly including kinetic quantum sieving (KQS) and chemical affinity quantum sieving (CAQS). Recent experimental advances in the separation of hydrogen isotopes from MOFs and zeolites are highlighted.

## 1. Introduction

Hydrogen is the simplest and most abundant element in the universe and is considered to be the most promising clean energy source for the future. Deuterium–tritium fusion reactor is a large-scale device that uses two heavy isotopes of deuterium (^2^H or D) and tritium (^3^H or T) as high-temperature plasma fuels and conducts fusion nuclear reactions under the confinement of a superconducting magnetic field to emit large amounts of energy [1,2]. Hydrogen isotopes are widely studied as energy carriers for the nuclear industry. In addition, isotope effects have important applications in neutron scattering [3,4,5], medicine [6], and isotope tracing [7]. However, due to their low natural abundance (D_2_: 0.015%, T_2_: ~6.7 × 10^18^%), they are efficiently recovered from fission reaction wastes to meet economic and environmental safety requirements [8]. Their physical and chemical properties are very similar (molecular dynamics diameter is the same at room temperature: 2.89 Å, boiling point close to 20.2–25.0 K), resulting in its efficient separation being extremely difficult [9]. At present, the separation methods commonly used in industry are mainly low-temperature rectification and Girdler curing process. Low-temperature distillation is mainly based on the different boiling points of protium (20.2 K), deuterium (23.7 K), and tritium (25.0 K), and the separation is achieved by multi-stage distillation column, which has the advantages of good separation effect, but it has problems of high cost, complex operation, and high energy consumption. In Girdler’s vulcanization process, deuterium and tritium are extracted by exchange reaction according to the different equilibrium constants of different hydrogen isotopes and hydrogen sulfide in water at different temperatures. At low temperatures, hydrogen sulfide is exchanged with deuterium and tritium in water to form D_2_S and T_2_S, which are re-decomposed to form D_2_O and T_2_O at high temperatures, then the corresponding D_2_ and T_2_ are produced by electrolysis. This method not only has high energy consumption and cannot reach the ideal separation ratio (D_2_/H_2_ separation ratio is only 1.3), but also the use of hydrogen sulfide is easy to cause equipment corrosion, and its flammability brings safety risks to industrial production [10]. High separation factors and high natural exchange rates are the main desired properties for HIS (HIS). Unfortunately, hydrogen isotopes have almost identical physical properties, making their separation difficult with conventional molecular sieves [11], whereas quantum sieving (QS) as an emerging separation technique has been shown to be remarkably effective in the separation of hydrogen isotopes. The concept of QS was first introduced in 1995 by Beenakker et al. [12]. QS has very stringent pore requirements for the separating material, and the QS effect can only occur when the difference (d-σ) between the gas molecule size (σ) and the pore size (d) of the microporous material is close to the De Broglie wavelength (λ) of the gas molecules. The de Broglie wavelength, also referred to as the wavelength of matter waves, serves as a pivotal concept in quantum mechanics, encapsulating the dual nature of particles exhibiting both wave and particle characteristics. Initially introduced by French physicist Louis de Broglie in 1924, this concept offers an explanation for the wave-like properties exhibited by microscopic particles, including electrons. The de Broglie wavelength quantifies the wave-like attributes of particles, highlighting how their momentum influences their oscillatory behavior in space. The computation of the de Broglie wavelength relies on the formula λ = h/p, where λ represents the wavelength, h denotes the Planck constant with an approximate value of 6.626 × 10^−34^ Js, and p signifies the momentum of the particle. Momentum is determined by multiplying the mass (m) of the particle by its velocity (v), expressed as p = mv. Notably, the de Broglie wavelength underscores a fundamental principle: as the momentum of a particle decreases (due to a smaller mass or slower velocity), its wavelength increases, resulting in more pronounced wave-like characteristics. This concept holds significant importance in elucidating quantum phenomena, such as quantum tunneling and electron diffraction, and forms a cornerstone of quantum mechanics. Furthermore, in the context of quantum sieving for HIS, the de Broglie wavelength plays a crucial role, as it governs the transport behavior of hydrogen isotopes within nanopores. Therefore, researchers have explored aperture control, flexible holes, gated opening, and other methods to enhance KQS. Chemical affinity quantum sieve (CAQS) is an alternative to KQS-based HIS of microporous materials at low temperatures. The key to achieving higher separation factors with CAQS is to increase the difference in the enthalpy of adsorption at the adsorption site with respect to H_2_/D_2_.

This paper provides a brief overview of the principles of QS, focusing on a review of design strategies for developing and improving various materials for HIS and the reasons for their highly selective formation. Most of the material designs are related to different metal atom radius sizes, metal sites, and metal hydrogen uptake capacity. The aim of this paper is to summarize the development and design ideas of these materials and provide a valuable way to find ideal HIS materials.

The low-temperature distillation solidification process serves as an industrial technique for separating hydrogen isotopes, including H_2_ and deuterium gas (D_2_). This process relies on the subtle differences in physical and chemical properties exhibited by hydrogen isotope molecules, particularly their boiling points, with H_2_ boiling at 20.3 K and D_2_ boiling at 23.7 K. The general steps involved in the low-temperature distillation solidification process are outlined as follows: Initially, a cooling process is employed to chill the gas mixture containing hydrogen isotopes to temperatures approaching absolute zero, typically hovering around 20 K, to facilitate the liquefaction of the gases. Subsequently, an evaporation process takes place. At these extremely low temperatures, lighter H_2_ evaporates more easily compared to deuterium gas (D_2_) due to their differing boiling points. The separation process is achieved by meticulously controlling the evaporation, ensuring that the evaporated gas contains a higher proportion of H_2_ than D_2_, thus achieving a preliminary separation of the two isotopes. This is followed by condensation and circulation processes. Lastly, purification is carried out. However, a significant drawback of this distillation solidification process is its requirement for substantial energy to sustain the extremely low operating temperatures, coupled with relatively low separation efficiency (for instance, the separation factor for D_2_/H_2_ is approximately 1.5 at 24 K). Additionally, the potential for equipment blockage due to the presence of water vapor necessitates the removal of water vapor from the gas mixture prior to the cooling process. These factors contribute to the high cost and energy consumption associated with the industrial applications.

Therefore, researchers are continually exploring more efficient and cost-effective isotope separation technologies, such as leveraging the quantum sieving effect of MOFs and zeolite materials.

## 2. KQS and CAQS Mechanisms

**Figure 1:**
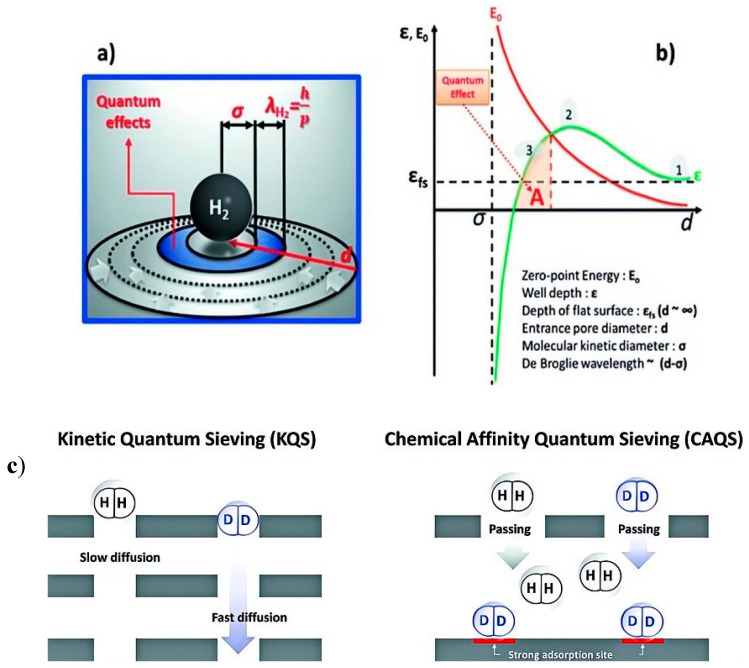
(**a**) Schematic representation of the quantum confinement effect. (**b**) Behavior of the well depth (*ε*) and zero-point energy (*E*0) as a function of channel diameter. (**c**) Adsorption potential depth as a function of pore diameter. (**c**) QS mechanism: KQS and CAQS [13].

KQS occurs when the difference between the kinetic diameter of a molecule and the size of its pores is at the de Broglie wavelength [12]. Figure 1a,b present schematic illustrations of quantum confinement effects, encompassing the variation of zero-point energy (E0) and adsorption potential depth (ε) of molecules confined in pores as a function of channel diameter, along with the changes in adsorption potential depth with pore size [13,14]. In Figure 1b, ε is also depicted as a function of aperture (i.e., molecules in the slit channel), starting from its value on a flat surface (ε fs). For example, when d is large enough, the potential energy relative to the wall does not affect each other and can be approximated as two independent flat surfaces, denoted as ε fs (marked as 1 in Figure 1). As the hole width decreases, this potential energy overlap becomes stronger and stronger until the minimum values of potential energy relative to the wall come into contact with each other (marked as 2 in Figure 1). If d is further reduced, the potential depth begins to decrease (marked as 3 in Figure 1), and finally when d is the same or smaller than σ, the molecule can no longer physically penetrate the pore. It should also be noted that before reaching d = σ, ε becomes negative (Figure 1b), indicating that in pores slightly larger than the molecule, electrostatic repulsion between the electron shell between the pore and the molecule has occurred, making adsorption difficult. These diagrams facilitate comprehension of the intricate relationship between adsorption enthalpy and pore size, as well as the profound influence of quantum effects on the adsorption and separation of hydrogen isotopes within MOFs [15,16]. At this point, the radial motion of the molecules is restricted, and the separation of the hydrogen isotopes is determined by the diffusion rate [17,18,19]. When the pore diameter of the porous material is equivalent to the de Broglie wavelength of the light isotope, the quantum effect allows the separation of heavier molecular species based on the difference in diffusion rate (the diffusion rate of the heavier isotope is faster than that of the lighter isotope) [20]. It is worth noting that this quantum effect is particularly prominent at low temperatures where the de Broglie wavelength difference is large. This difference in diffusion rates between H_2_ and D_2_ (D_2_ has high mobility in pore media, while H_2_ has low mobility) is utilized to achieve separation, as shown in Figure 1c. The KQS separation mechanism depends mainly on the pore structure of the material and the kinetic nature of the hydrogen molecules. Striking a balance between selectivity and adsorption capacity is challenging because pore size limitation increases the selectivity of gas separation while decreasing the adsorption capacity.

The CAQS effect used for the separation of hydrogen isotopes is based on the principle that the different masses of hydrogen isotopes, the zero-point energies (ZPEs) of the strong adsorption sites adsorbed on the surface of the nanoporous material, are different, which leads to a change in the enthalpy of adsorption. The strength of adsorption is mainly related to the zero-point energy. The heavier hydrogen isotopes D_2_ have higher binding enthalpies and thus bind preferentially to the adsorption sites [21,22]. As shown in Figure 1c, molecules of large-mass D_2_ have a strong binding capacity to the adsorption site and are thus bound to the adsorption site, leading to the separation of hydrogen isotopes. In addition, since this separation is achieved by adsorption of isotope gases, the process is not limited by pore size [23]. As shown in Figure 1c, molecules of large-mass D_2_ have a strong binding capacity to the adsorption site and are thus bound to the adsorption site, leading to the separation of hydrogen isotopes. Additionally, it is noteworthy that CAQS can separate hydrogen isotopes even at higher temperatures (≥77 K). At present, there are already materials that can achieve a higher separation ratio above 77 K, which will make hydrogen deuterium separation materials through quantum screening meet the needs of practical industrial production. Therefore, the design of strong adsorption sites will be the key to prepare efficient HIS gas materials.

The integration of KQS and CAQS promises to enhance the separation coefficient. However, when attempting to merge these two mechanisms, a crucial consideration arises: if the material is specifically optimized for KQS, which is governed by pore size, it may not attain optimal performance when the focus shifts to CAQS, which relies heavily on surface chemistry. This is exemplified by the study conducted by Hu et al. on Cu(I)Cu(II)-BTC, a material utilized for the separation of a 1:1 hydrogen–deuterium mixture. Their research revealed that the unique Cu(I) and Cu(II) coordination network inherent in Cu(I)Cu(II)-BTC significantly facilitates D_2_/H_2_ isotope separation. Nevertheless, a limiting factor emerges: the dz2 orbitals and positive charges of Cu(I) are unable to effectively engage in hydrogen interactions, resulting in a comparatively weak hydrogen adsorption and binding strength. Consequently, this material fails to exhibit the desired high chemical affinity effects of Cu(I) sites at temperatures above liquid nitrogen, thereby compromising its effectiveness in HIS. This case underscores the potential benefits of combining KQS and CAQS but also highlights the challenges that may arise in practical applications due to specific structural characteristics. These challenges, including temperature dependence, the delicate balance between pore size and adsorption sites, material synthesis and stability, and cost and scalability considerations, must be addressed through interdisciplinary research and innovation. Only by doing so can we hope to achieve more efficient and cost-effective HIS technologies [23].

## 3. MOFs

Before introducing previous studies, the summary of them based on the highest separation factor is presented to help readers’ understanding (Table 1).

### 3.1. KQS for Hydrogen Isotopes by MOFs

MOFs are a class of porous material formed by metal ions or metal clusters connected with organic ligands through covalent bonds. They show great potential for applications in gas adsorption, separation, and storage, especially in the field of HIS, due to their high specific surface area, tunable pore size and designable chemical functionality. In 2008, Chen et. al. observed for the first time the adsorption kinetic constant of D_2_ significantly faster than that of H_2_ with lower activation energy at 77 K using Zn-MOF material (whose pore size is <5.6 Å) [24]. They suggest that this is a QS effect due to the larger zero-point energy of H_2_ due to the difference in vibrational energy levels of the quantum statistical mass effect. This was the first time that the QS effect was observed experimentally, thus giving rise to a wave of research in KQS.

**Figure 2:**
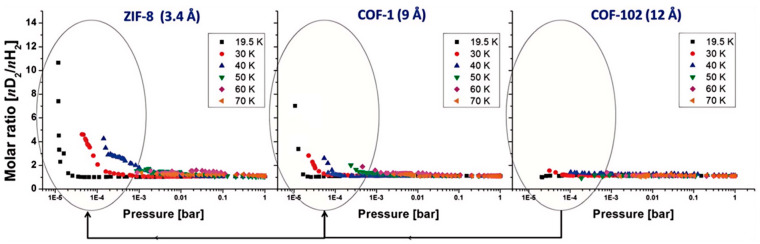
Comparison of the molar ratio (nD_2_/nH_2_) as a function of the effective pore size of organic frameworks in the temperature range of 19.5–70 K and a pressure range of 0–1 bar [25].

In order to elucidate (or maximize) these quantum effects, precise control of the pore size is an essential prerequisite. The nature of MOFs to enable pore size tuning through the selection of different organic ligands and metal ions has led to a wider study of MOFs for KQS in HISs. Oh et al. investigate the H_2_ and D_2_ adsorption properties of different microporous framework materials (ZIF-7, ZIF-8, COF-1, and COF-102) at low temperatures (below 77 K) and determine the KQS in correlation with pore size [25]. As shown in Figure 2, they find that there is a maximum molar ratio when the pore diameter is reduced to 3.4 Å, and some experimental results clearly show that the optimal pore size for QS has to be larger than 3.0 Å. Therefore, they conclude that the optimum diameter of the aperture should be in the range of 3.0–3.4 Å in a rigid frame. Mondal et al. investigated a series of zinc-based isostructural imidazolate frameworks, specifically IFP-1, IFP-3, IFP-4, and IFP-7, which had one-dimensional channels with different pore sizes [26]. The pore sizes of IFP-1 and IFP-3 are slightly larger than the kinetic diameter of the hydrogen isotopes and are suitable for quantum screening, with IFP-3 having a smaller pore size and the highest selectivity of about S = 2.5. The lower selectivity is due to the fact that the small pore volume prevents the passage of the hydrogen isotopes within the 1D channel structure, and the QS occurs only in the outermost pore aperture. Thus, the ultra-microporous material with narrow 1D channels results in poor selectivity for QS. Their study proposes that in order to achieve effective QS with high selectivity, two fundamental structural properties are required: (1) a small pore size for quantum screening and (2) a range of pore diameters and a large pore volume that allows isotope molecules to be exchanged inside the material and to undergo multiple QS at each pore opening.

**Figure 3:**
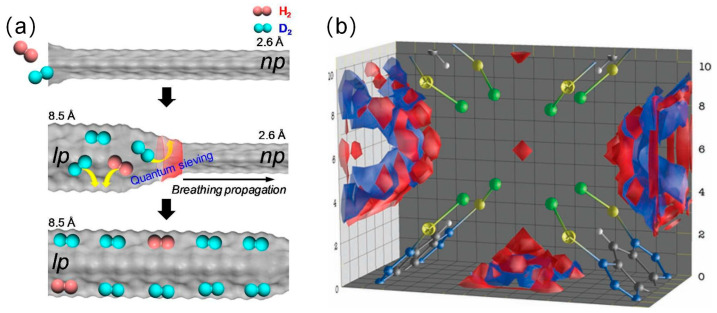
(**a**) Schematic view of D_2_ separation in breathing propagation along the 1D channel in MIL-53(Al) [14] (**b**) Local density differences n(D_2_)-n(H_2_) in a box with the same number of D_2_ and H_2_ particles. The red area in the middle indicates an excess of D2 near the cavity surface and aperture; Yellow represents zinc atoms, green represents chlorine atoms, gray represents carbon atoms, and blue represents nitrogen atoms. The red areas indicate a D_2_ excess close to the cavity surface and the aperture; the blue areas indicate a H_2_ excess inside the cavities [29].

Structural flexibility and dynamic properties allow for localized deformation and thus exchange within the material and multiple quantum screening at each orifice. Benefiting from advances in porous coordination polymers research [27,28], the emergence of flexible MOFs has enabled direct dynamic pore size for maximum separation factor. In a subsequent study, Kim et al. reported for the first time a strategy for efficient separation of hydrogen isotopes using dynamic changes in pore size in MIL-53(Al) [14]. As shown in Figure 3a, considering the dynamic pore changes, MIL-53(Al) definitely produces intermediate apertures during the phase transition from large to small pores, i.e., respiration of one-dimensional channels. This intermediate pore size obtained by working at a given temperature and pressure allows for optimal quantum interactions between D_2_ and the framework, whereas the interaction between H_2_ and the framework remains weak, thus maximizing the KQS effect. MIL-53(Al) achieves a selectivity of 10.5 and an adsorption capacity of 2.3 mmol/g at 40 k. Also, controlling the pressure and achieving multiple QSs can be achieved by localized deformation instead of an overall change in pore volume. Teufel et al. investigated a zinc-based MFU-4 with alternating small- and large-cavity pore systems, as shown in Figure 3b [28]. MFU-4 can be regarded as a small cavity acting as a separation gate due to its unique bimodal pore structure, while the large cavity provides a surface area for storing a large amount of D_2_, resulting in a maximum selectivity of 7.5 for H_2_/D_2_.

Bondorf et al. found the gating effect to be an efficient method for isotope separation in their study of flexible MOF materials with DUT-8(Ni) [30]. They demonstrated that the MOF remains closed when DUT-8(Ni) was exposed to H_2_. The same was observed when DUT-8(Ni) was exposed to the mixed isotope HD. In contrast, the gate opened easily between 23.3 and 26 k when exposed to D_2_. The similarity between the D_2_ partial pressure response of the pure D_2_ gas and that of the gas mixture demonstrates that the DUT-8(Ni) phase transition was driven by the D_2_ partial pressure and not by the total gas pressure. As a result of this selection, DUT-8(Ni) realized a D_2_/H_2_ selectivity of 11.6 at 23.3 K.

**Figure 4:**
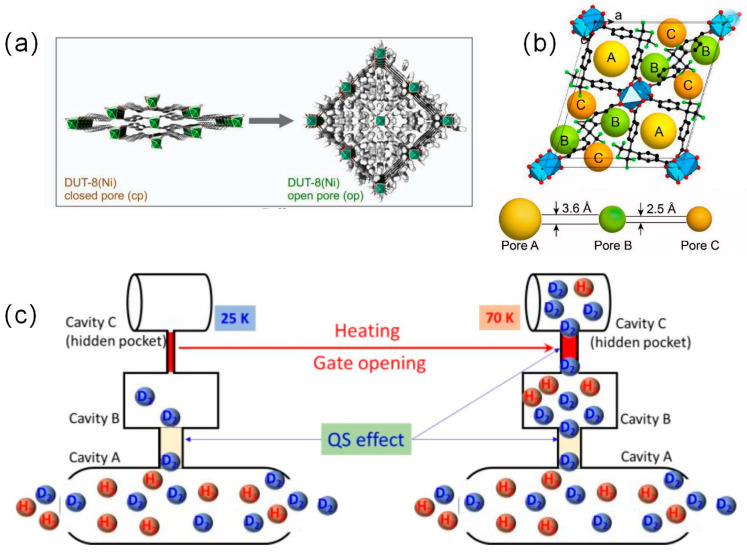
(**a**) Crystal structure of DUT-8(Ni) in cp and D_2_@DUT-8(Ni) op phases [30] (**b**) Unit cell and crystal structure of FMOFCu. The yellow, green, and brown spheres represent the volume of pores A, B, and C, respectively [31]. (**c**) Schematic view of the trimodal structure of FMOFCu [31].

Zhang et al. found high enthalpies of physical adsorption due to the exposure of fluorine atoms on the pore surface and the small pore size of the structure. FMOFCu (partially fluorinated metal–organic framework containing copper) studied by Zhang et al. is a porous material with a three-modal pore system that combines most of the features described above, as shown in Figure 4c. The unique pore structure and temperature-dependent gating effect of FMOFCu can effectively separate hydrogen isotopes [31].

**Figure 5:**
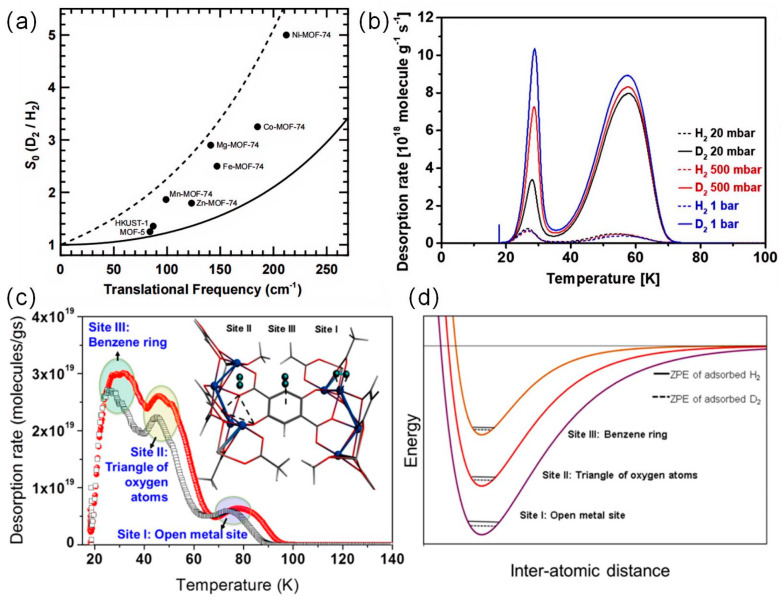
(**a**) Zero-pressure selectivity of MOF materials at 77 K versus infrared translational mode frequencies [23]. (**b**) TDS of CoFA measured at a heating rate of 3 K/min [33]. (**c**) TDS of D2CPO-27-Co. Inset: identification of three adsorption sites on CPO-27-Co. (**d**) Qualitative plot of adsorption site intensities. Site I is a metal site, site II is a triangle of oxygen atoms, and site III is a benzene ring [34].

Hulvey et al. reported for the first time hybrid materials containing perfluorinated ligands and showed unique interactions [32]. FMOFCu exhibits alternating small and large cavity structures and hidden chambers blocked by fluoride windows in the chamber structure, which would be favorable for enhanced KQS. They observe that the restricted pore size of 3.6 Å at 25 K creates a strong diffusion barrier, which allows for preferential penetration of D_2_ into cavities A and B with high selectivity. As the temperature increases to 40 K, the increase in isotopic absorption and decrease in selectivity is related to localized vibrations opening the aperture and sufficient kinetic energy of the molecule. When the temperature is greater than 60 K, a third desorption peak appears, showing thermally activated opening of the 2.5 Å aperture connecting the cavities C, as shown in Figure 5d. The selectivity of FMOFCu for D_2_ reaches 14. At the same time, the material demonstrates high desorption temperatures achieved by KQS in the absence of an open metal site.

### 3.2. CAQS for Hydrogen Isotopes by MOFs

**Figure 6:**
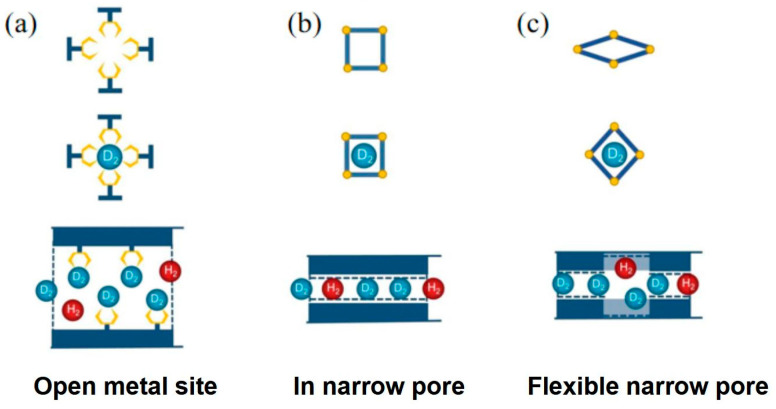
Three structures that can increase the actual temperature of HIS: (**a**) open metal sites; (**b**) fine one-dimensional channels; and (**c**) flexible narrow pores [36].

The CAQS proposed by FitzGerald provides a method for the separation of hydrogen isotopes at relatively high temperatures (equal to or above the temperature of liquid nitrogen) as an alternative to KQS-based HIS of microporous materials at low temperatures. The heavier isotope D_2_ has a lower zero-point energy, which leads to a difference in the enthalpy of adsorption, thus allowing the main adsorption to be on D_2_. The key to achieving higher separation factors lies in increasing the enthalpy of adsorption at the adsorption site. FitzGerald et al. observe that the selectivity in the Ni-MOF-74 material is 5.0 at 77 K and decreases to 1.5 at 150 K [23]. As shown in Figure 6a, selectivity is strongly correlated with the adsorbed H_2_ advection frequency based on IR measurements. This confirms that the separation is mainly due to the difference in zero-point energies of the adsorbed isotopes. CAQS exhibits a higher desorption temperature unlike KQS for the separation of hydrogen isotopes. Designing MOFs with numerous strong binding sites significantly improves the overall isotope separation performance even at high temperatures. Oh et al. identify the preferred adsorption sites on CPO-27-Co and estimate the adsorption strength of these sites by low-temperature thermal desorption spectroscopy (TDS) [35]. As shown in Figure 6c,d, they demonstrate that the low-energy adsorption sites II and III on CPO-27-Co exhibit weak ZPE differences and therefore poor selectivity. Additionally, at temperatures higher than 50 K, only the open metal site Co(II) remains occupied. The strong attraction between the unsaturated Co(II) center and hydrogen keeps these sites occupied at relatively high temperatures, and the isotope effect is strong enough to lead to enthalpy differences over a wide range of temperatures. The separation selectivity of CPO-27-Co for H_2_/D_2_ at 60 K reaches 12. Weinrauch et al. investigated the ability of MOFs containing active Cu(I) sites to capture heavy hydrogen isotopes from hydrogen by selective adsorption [16]. Cu(I)-MFU-4l exhibited a remarkable isotope effect with excellent enthalpy of adsorption and selectivity, even at temperatures higher than 100 K. Cu(I)-MFU-4l achieve D_2_/H_2_ selectivity of 11 at 100 K.

Muhammad et al. studied a MOF material called cobalt formate (CoFA), a diamond-like framework with one-dimensional open zigzag channels [33]. They found a single desorption signal for D_2_ at high temperatures indicative of adsorption at the first and second sites and a strong desorption peak (third site) at low temperatures due to densely stacked D_2_ adsorption, as shown in Figure 5b. This third adsorption site responds only to D_2_ and originates from the enlargement of the internal cavity due to D_2_ adsorption.

The results led to a significant difference in D_2_ and H_2_ adsorption, with CoFA showing up to 44 D_2_/H_2_ selectivity and 7 mmol/g D_2_ adsorption at 25 K. Si et al. investigated FJI-Y11 with the rare chabazite (CHA) zeolite topology [37]. They found that the adsorption sites of FJI-Y11 are not open copper sites exposed on the paddlewheel clusters but oxygen atoms on the ligands, which play an important role in the adsorption of D_2_. The adsorption capacity of FJI-Y11 for D_2_ can reach 205 cm^3^/g at 77 K and 1 bar and shows good regeneration ability and structural stability.

### 3.3. The Synergistic Effect of KQS and CAQS for Hydrogen Isotopes by MOFs

Three pore structures for efficient separation of hydrogen isotopes are illustrated in Figure 6: (a) open metal sites; (b) fine one-dimensional channels; and (c) flexible narrow pores. The simultaneous invitation of KQS and CAQS is favorable for enhancing the selectivity. Kim et al. chose the MOF-74 system with a high enthalpy of hydrogen adsorption, in particular the MOF-74-Ni [17].

The MOF-74-Ni sample is subjected to different post-treatments to introduce different amounts of imidazole molecules to modulate the pore size and repeatedly prevent hydrogen diffusion, thereby enhancing KQS. By optimizing the amount of imidazole molecules, the synergistic effect of KQS and CAQS effects can be achieved simultaneously, resulting in high selectivity up to 26 while maintaining a high D_2_ adsorption of 2.84 mmol/g. This method of introducing imidazole molecules via a post-synthesis strategy not only avoids the need for complex design and synthesis of new MOF structures but is also applicable to the separation of other gas mixtures. Hu et al. synthesize HKUST-1-derived microporous mixed-valence Cu(I)Cu(II)-BTC material [38]. The introduction of Cu(I)OMS, a material with a unique network of Cu(I) and Cu(II) coordination sites, into the framework can significantly enhance the D_2_/H_2_ isotope separation. Their results indicate that the appearance of geometrically characterized structures of Cu(I) sites can reduce the pore size and increase the hydrogen adsorption strength of Cu(II) sites. The selectivity (D_2_/H_2_) of Cu(I)Cu(II)-BTC for D_2_/H_2_ reached 37.9 at 30 K, which is attributed by them to the synergistic effect of KQS and CAQS.

### 3.4. Search for Comfortable MOFs by Machine Learning

Based on the development of machine learning, high-throughput searches for materials can be more efficient in finding materials with ideal properties [39]. Chen et al. conducted a study combining a database of experimentally reported MOFs and a newly constructed database of MOFs, applying machine learning and feature engineering to predict and evaluate the performance of MOFs for the separation of hydrogen isotopes. Their study identified 33 MOFs with high overall performance indices and 12 MOFs with high D_2_/H_2_ selectivity. This multiscale study provides new insights into the application of MOFs in the field of HIS systems and demonstrates that machine learning methods can not only accurately and efficiently reveal key material properties but also accelerate the discovery of high-performance adsorbents.

In Musen Zhou et al.’s study, machine learning was employed for high-throughput screening of 12,723 experimentally synthesizable MOF (metal–organic framework) membranes in QS and D_2_/H_2_ separation, aiming to predict their gas adsorption and transport performance in D_2_/H_2_ separation. This approach enables the rapid identification of MOF materials possessing optimal selectivity and capacity. Furthermore, machine learning facilitates membrane performance scoring (MPS) and pinpoints the structural features of MOF membranes exhibiting high D_2_/H_2_ separation efficiency. In the article, various machine learning models, including Support Vector Machine (SVM), Random Forest (RF), Gradient Boosting Tree (GBT), and Deep Neural Network (DNN), were utilized for both classification and regression analysis of MOF material performance. These models are adept at pinpointing MOFs with the top 10% ideal membrane selectivity. Specifically, machine learning models, SVM foremost among them, are employed to predict the structural characteristics of MOFs with optimal membrane selectivity. These predictive outcomes aid in comprehending the influence of PLD and LCD on D_2_/H_2_ separation efficiency, thereby guiding the design of more efficient MOF structures. These instances illustrate that machine learning has played a pivotal role in MOF material design and HIS, not only enhancing the efficiency of screening and prediction but also furnishing fresh insights and guidance for material design. Through the application of machine learning models, researchers can swiftly identify materials with potential application value and optimize their structures to attain superior separation performance [40].

The adsorption enthalpy data of MOFs exhibit significant variations depending on the material type, temperature, and adsorbate. For instance, COF-102 demonstrates enthalpy and a hydrogen isotope effect selectivity of 1, superior adsorption lower temperatures. Conversely, Ni-MOF-74 possesses a higher adsorption enthalpy and a hydrogen isotope effect selectivity of 4.6, enabling it to maintain a high adsorption capacity even at elevated temperatures. The intricate relationship between adsorption enthalpy and hydrogen is influenced by numerous factors, interactions, quantum size effects, chemical affinity structural characteristics, and operating temperature. By meticulously controlling these parameters, it is possible to optimize the separation selectivity, ultimately facilitating efficient separation.

## 4. Zeolites

Before introducing previous studies, the summary of them based on the highest separation factor is presented to help readers’ understanding (Table 2 and Table 3).

### 4.1. KQS for Hydrogen Isotopes by Zeolites

**Figure 7:**
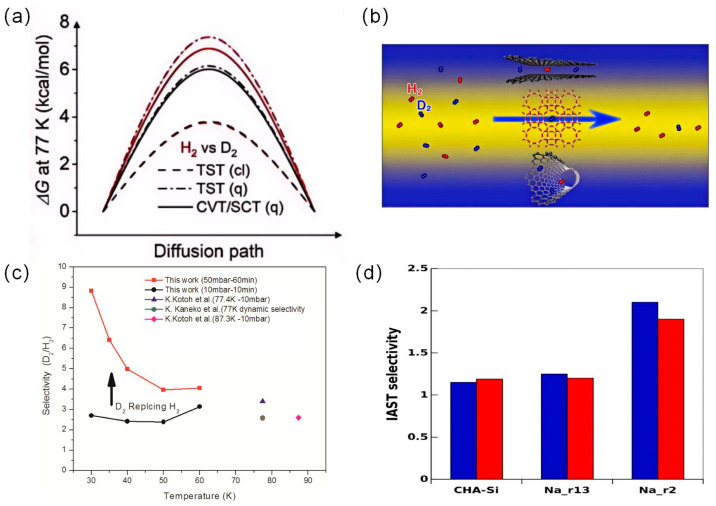
(**a**) Calculated base state vibrational adiabatic potential curves [4]. (**b**) MS13X cylindrical pore structure [45]. (**c**) Selectivity of D_2_/H_2_ under different exposure conditions [46]. (**d**) Selectivity at D_2_/H_2_ (25/75) 77.4 K. Na SI has the largest Si/Al; NaK_r2 has a Si/Al ratio of 2.1; and Na_r13 has a Si/Al ratio of 13 [47].

Zeolite is a crystalline aluminum silicate material. It is named “zeolite” because of the boiling phenomenon that occurs when it is burned. Their Si-O and Al-O tetrahedral structure allows them to form sub-nanopores (≤1.0 nm) within their framework. Gao used canonical variational theory and small curvature tunneling contributions to investigate the effect of quantum effects on H_2_ diffusion in pure silica zeolites RHO [41]. As shown in Figure 7a, they found an anomalous kinetic isotope effect (KIE) at low temperatures, i.e., the heavier isotope species D_2_ diffuses faster than the lighter isotope H_2_. This anomalous KIE is mainly caused by differences in ZPE, with the lower ZPE of D_2_ leading to its smaller effective potential barrier during tunneling. The nanopore size (e.g., pore size, window size) of the close isotopic species is crucial for determining the KQS effectiveness of zeolites. Zeolites have a very uniform pore size, which can be adjusted to the optimum pore size by choosing the right type of zeolite. Chu et al. measured the adsorption equilibrium of H_2_ and D_2_ on SBA-15, 5A, Y, and 10X molecular sieves at 77 K using the volumetric method [42]. The equilibrium adsorption ratio (R_D/H_) of D_2_ to H_2_ on SBA-15 mesoporous molecular sieves was greater than that on microporous molecular sieves (5A, Y, and 10X), but the difference between adsorbents decreased with increasing pressure. The pore size of the molecular sieve has a significant effect on the adsorption and separation of hydrogen isotopes. Smaller pore size molecular sieves are more favorable for the separation of hydrogen isotope mixtures. Kotoh studied synthetic zeolites 4A (SZ-4A) and 5A (SZ-5A) as adsorbents [43]. They found that the pore size decreased from 5 Å to 4 Å and the selectivity increased from 2.8 to 3.4 at 77 K and 1 kPa. Kotoh et al. investigated the adsorption behavior of hydrogen isotope molecules on synthetic zeolites 4A, 5A, and 13X at liquid nitrogen temperature (77.4 K) [44]. They found that the molecular sieving phenomenon occurred in the temperature range above 77.4 K, which was interpreted as a result of the variation of the pore size of the molecular sieves with temperature.

Niimura et al. determined the separation coefficients of molecular sieves such as MS4A, MS5A, MS13X, RHO, and H-ZSM-11 at 77 K and 5 mbar [45]. The results showed that MS13X had the highest separation coefficient of 3.05 at 77 K compared to the other materials. As shown in Figure 7b, the authors attributed this result to its interconnected cylindrical pore structure, which resulted in a significant diffusion hindrance to H_2_. Xiong et al. reported hydrogen isotope selectivity of 5A zeolites at temperatures below 77 K [46]. The selectivity of D_2_/H_2_ increased gradually with loading time and pressure and reaches a constant value. As shown in Figure 7c, it is 8.83, 6.42, 4.98, 3.96, and 4.04 at 30, 35, 40, 50, and 60 K, respectively (at 50 mbar). Bezverkhyy studied pure silica and sodium-containing CHA zeolites with different Si/Al ratios, as well as zeolites with mixed K-Na and Li contents [47]. At 77.4 K, the adsorption capacity of CHA zeolite increased with increasing aluminum content until the Si/Al ratio was 2.1, as shown in Figure 7d. They found that the cation composition had less effect on the D_2_/H_2_ selectivity, but the selectivity increased exponentially with decreasing temperature.

### 4.2. CAQS for Hydrogen Isotopes by Zeolites

**Figure 8:**
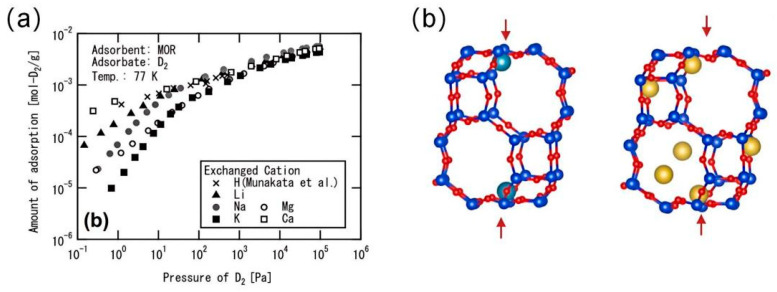
(**a**) MOR adsorption isotherms for different cations at 77 K [48]. (**b**) Schematic representation of cation positions in the structural cage of CHA on MOR: Ca-CHA and Na-CHA. (Atomic colors: red—O, dark blue—Al or Si, light blue—Ca, and yellow—Na, The arrows indicate the 6MR windows [50]).

Molecules with a strong chemical affinity for the surface in the pores are usually adsorbed, whereas molecules with weak interactions are not. D_2_ can be preferentially adsorbed on strong binding sites of the host material, mainly unsaturated metal centers, by chemical affinity screening. Due to the lower ZPE, the heavier isotopes interact more strongly with the adsorbent, leading to higher enthalpies of adsorption (ΔH). Kawamura synthesizes a mercerized zeolite-type zeolite (Na-MOR) as a starting material and replaces sodium ions (Na^+^) therein with alkali metal ions or alkaline earth metal ions (e.g., Li^+^, K^+^, Mg^2+^, and Ca^2+^) by a cation-exchange reaction [48]. As shown in Figure 8a, cation exchange significantly affects the adsorption capacity in the low-pressure range of 77 K. In particular, the adsorption capacity of alkali metal ions (monovalent cations) seems to increase with decreasing atomic number. Giraudet synthesized a series of FAU-type zeolites that are substituted by different cations (Li^+^, K^+^, Mg^2+^ and Ca^2+^, Ba^2+^ and Mn^2+^) [49]. In addition, CHA and MFI zeolites of NaY, DAY (de-aluminized Y), and pure silica have been studied. They found that the selectivity of exchanged zeolite X at low loadings decreases with increasing cation size, from 5.7 for MgX to 2.4 for KX. At high loading, the selectivity was not affected by material composition and was about 1.5 for all materials studied. For cation-free zeolites (CHA and MFI), the latter value was observed at all loadings. They suggest that the key role of cations in D_2_/H_2_ selectivity is through adsorption via strong guest–cation interactions at low loadings, while weak interactions are involved at high loadings.

Bezverkhyy et al. studied two zeolites with high Al content, Na-CHA and Ca-CHA (Si/Al = 2.1) [50]. Na-CHA showed a selectivity of 25.8 with an adsorption capacity of 10.6 mmol/g at 38 K. At the same temperature, Ca-CHA showed a slightly lower selectivity (18.3) but a higher adsorption capacity (12.9 mmol/g) than that of Na-CHA. They found that the D_2_/H_2_ selectivity is not only dependent on the size of the window and the strength of cation–guest interactions but also may be associated with the eight-membered ring window (8MR) to form new adsorption sites at high loadings. It is demonstrated that the cations in Ca-CHA only occupy positions within the cage near the top and bottom six-membered ring (6MR) windows. In contrast, in Na-CHA, four of the six 8MR windows are occupied to form new adsorption sites, leading to higher selectivity. In another study, Bezverkhyy et al. investigated the effect of partial Na replacement by K on D_2_/H_2_ adsorption on LTA zeolite at 77 K [51]. The 12th cation in Na-LTA zeolite is located near the four-membered ring (4MR). Changing the cation in the pore window can regulate the size of the pore window, thereby controlling the pathway of molecules into the α-cage. The pore size of the sodium form of LTA is ~4 Å, whereas partial exchange with potassium results in a reduction of the effective pore size to ~3 Å. In the K_1.6_Na_10.4_A zeolite, the D_2_/H_2_ selectivity was significantly higher than that of the NaA zeolite at 77 K due to the fact that a part of the α-cage is accessible only to the D_2_ molecule. The D_2_/H_2_ selectivity of K_1.6_Na_10.4_A reached 23 compared to only 8.5 for NaA for a 25% D_2_ + 75% H_2_ mixture at 48 K. Their results suggested that the performance of LTA zeolites in D_2_/H_2_ separations can be significantly affected by varying the cationic composition of the zeolite.

**Figure 9:**
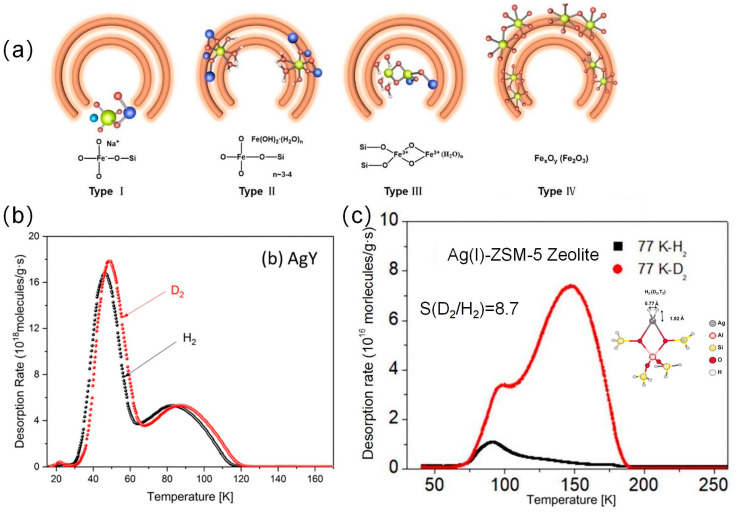
(**a**) Possible Fe-O coordination forms in e/ZSM-5 zeolite (green: Fe, red, O, sky blue: Si, pink: H, light blue: Na) [52]. (**b**) TDS curves of AgY zeolite after exposure to 10 mbar pure H_2_ and D_2_ at room temperature [53]. (**c**)TDS of Ag(I)-ZSM-5 at 10 mbar H_2_/D_2_ of 1:1 [54].

The ideal zeolite for HIS should have a considerable density of strong adsorption sites that preferentially attract D_2_ even at high temperatures. Although zeolites usually have cations that balance the negative charge of Al ions, these cations may not have a dominant effect on CAQS. Zhang et al. prepared Fe/ZSM-5 molecular sieves by ion-exchange method with D_2_/H_2_ selectivity of 32.1 [52]. They showed the presence of four Fe species in Fe/ZSM-5, of which the Fe-O cluster is directly related to the gas selectivity, as shown in Figure 9a. The Fe-O species acted as the main adsorption sites and interact more strongly with D_2_ than with H_2_. The D_2_/H_2_ selectivity increases with the increase in Fe content in Fe/ZSM-5. Zhang et al. studied the material Ag(I)Y (silver ion-exchanged molecular sieve Y) and found high gas adsorption due to the high density of silver sites resulting from the high Al content in the molecular sieve structure, with about one-third of the total physiorptive hydrogen isotopes adsorbed on silver sites (Figure 9b) [53]. D_2_/H_2_ selectivity was up to 10, and the maximum adsorption was 3 mmol/g at an exposure temperature of 90 K. Xiong investigated an HIS strategy based on strong chemical affinity quantum screening (CAQS) effect using Ag(I)-exchanged ZSM-5 zeolite [54]. Ag(I)-ZSM-5 zeolite enabled HIS above the liquid nitrogen temperature with high D_2_/H_2_ selectivity (8.7 at 77 K). By three adsorption/desorption cycles, 95.1% of D_2_ can be enriched from a mixture containing 2.5% D_2_. The high hydrogen isotope selectivity of Ag(I)-ZSM-5 zeolite was attributed to the strong chemical affinity at the Ag(I) site and the large isotope effect (Figure 9c).

It has been reported that due to the formation of H_2_ complexes, hydrogen molecules bind to active metal sites on the surface to form double hydrogen bonds [38,55]. This is best described as a combination of suitable empty metal d-orbitals positively energized by H_2_(σ) orbitals to the metal center and reverse energized by filled metal d-orbitals to the antibonding orbitals H_2_(σ*) [14,27]. In this interaction, the hydrogen bond is weakened, and the hydrogen bond length increases from 0.74 Å for free gaseous H_2_ to the usual 0.8~0.9 Å. The binding strength of the dihydrogen bond is intermediate between chemisorption and physisorption, which is typical of strong chemical affinity. Xiong et al. used commercial NH4-ZSM-5, MOR, and copper(II) acetate monohydrate to prepare Cu(I)-ZSM-5 and Cu(I)-MOR via a standard ion-exchange process [24]. The selectivity of Cu(I)-ZSM-5 for D_2_/H_2_ separation was as high as 24.9 at 100 K. They showed that Cu(I)-exchanged molecular sieves achieved efficient HIS and enrichment through strong chemical affinity between dihydrogen bonds and hydrogen isotopes. Three adsorption/desorption cycles allowed the enrichment of D_2_ from a mixture containing 2.5% D_2_ up to 99.6% D_2_ concentration, demonstrating the high efficiency and utility of Cu(I) exchanged molecular sieves for the separation and enrichment of hydrogen isotopes. In addition, Hu et al. used a synthesized HKUST-1-derived microporous mixed-valent Cu(I)Cu(II)-BTC (BTC stands for benzene-1,3,5-tricarboxylate) material with a unique network of Cu(I) and Cu(II) coordination sites [24].

**Figure 10:**
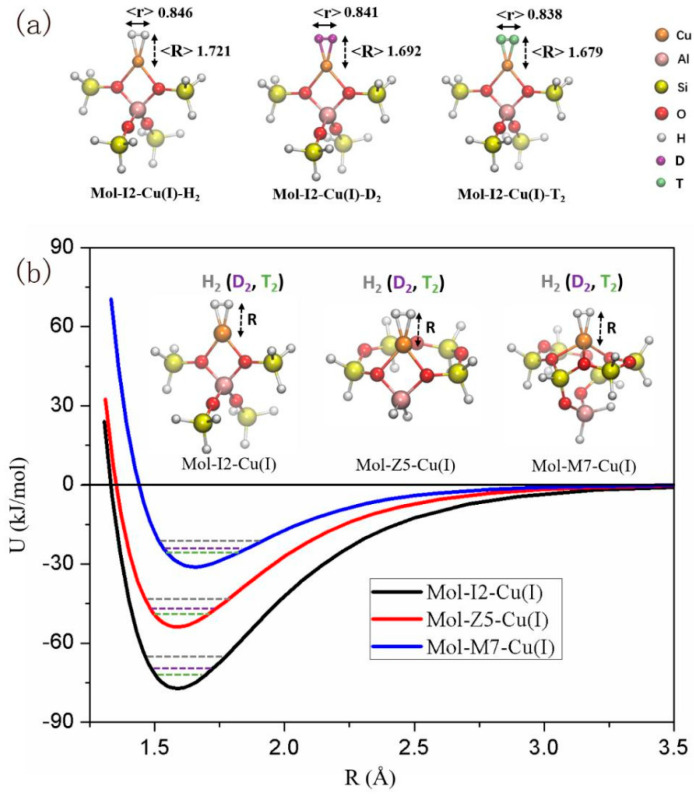
(**a**) Demonstrates that the anticipated distance and bond length between the heavier hydrogen isotopes (D_2_ and T_2_) and the Cu(I) center are both shorter than those with H_2_, suggestive of stronger interactions; (**b**) illustrates the interaction potential energy between various Cu(I) sites and hydrogen isotopes [24].

As shown in Figure 10, Hu et al. analyzed the adsorption behavior and separation mechanism of hydrogen isotopes in Cu (I)—ZSM-5 molecular sieve through DFT calculations [24]. To simplify the calculations, the researchers chose truncation units of Cu-ZSM-5 structure to investigate the interaction between H_2_ isotopes (H_2_, D_2_, T_2_) and the structure, with each truncation structure containing only one active metal ion (such as Cu (I)) [24]. Using Grimme’s DFT-D3 dispersion correction method to handle the empirical long-range contributions between atoms and molecules, harmonic vibration frequency analysis was performed on the optimized structure to obtain the zero-point energy (ZPE) of the system and the adsorption enthalpy of hydrogen isotopes. Finally, the expected distance between the adsorbed hydrogen isotope and the Cu (I) center was calculated using the Morse potential energy curve. According to the method described in the literature, the selectivity of isotopic molecules (H_2_, D_2_, T_2_, DH, TH, TD) on the Cu (I)—ZSM-5 structure was calculated.

In addition, the electron rearrangement between Cu(I) and adsorbed H_2_ was meticulously analyzed using the extended transition state method coupled with natural orbital chemical valence (ETS-NOCV), which revealed a profound interaction between the metal ions and H_2_. Furthermore, the hydrogen isotope selectivity of the Cu(I) center at the I2 site within Cu(I)-ZSM-5 was anticipated across various temperatures. Notably, the experimental outcomes aligned closely with the computational predictions, highlighting a decrease in D2/H_2_ selectivity as the temperature rises. Ultimately, DFT calculations corroborated the stronger interactions between heavier hydrogen isotopes, such as D_2_ and T_2_, and the Cu(I) sites. These interactions gave rise to significant disparities in zero-point energy and adsorption enthalpy, ultimately facilitating efficient isotope separation. The calculations revealed that at 77 K, the selectivity of S(D_2_/H_2_) and S(T_2_/H_2_) is exceptionally high, yet this selectivity diminishes with increasing temperature. This observation concurs with experimental findings, indicating that at lower temperatures, physical adsorption attenuates the overall isotope selectivity. In conclusion, DFT computational analysis offers theoretical insights into the robust chemical affinity, adsorption behavior, and separation mechanisms of hydrogen isotopes within Cu(I)-ZSM-5 molecular sieves. Additionally, it predicts isotope selectivity under diverse conditions, providing a comprehensive understanding of this complex system.

MOFs and zeolites have demonstrated promising potential in the realm of HIS, yet they encounter challenges pertaining to stability, scalability, and cost-effectiveness applications. To address these challenges, MOFs must retain their stability under high-temperature and high-pressure conditions while enhancing their scalability and structure–function relationships. On the other hand, a deeper understanding of zeolites’ properties is necessary to overcome their current limitations. The development of these materials necessitates innovative advancements in both experimental techniques and theoretical models, aiming to achieve more efficient and cost-effective HIS technologies.

## 5. COFs and POCs

**Figure 11:**
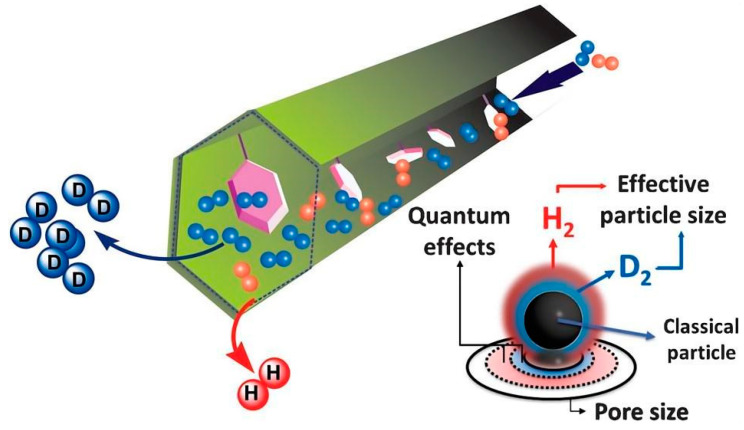
View of the pore channel of COF-1 with dangling pyridine molecules incorporated in the pore walls. The decreased size and the cryogenic flexibility of the aperture are employed for the enhancement of the quantum sieving effect for light isotopes [36].

COFs, the full name of which is Covalent Organic Frameworks, are materials characterized by ordered porous structures, formed by connecting organic monomers through covalent bonds to create two-dimensional or three-dimensional networks. These materials possess designable pore sizes and chemical functionality, which endows them with immense potential for applications in various fields such as gas storage, separation, catalysis, energy storage, and sensing. The distinctive features of COFs include high specific surface area, adjustable pore size, thermal and chemical stability, customizable chemical functionality, and lightweight nature. Researchers, Hyunchul and others, synthesized a novel material, Py@COF-1, by incorporating pyridine molecules (Py) into the pore walls of COF-1. This material demonstrates unique adsorption behavior at low temperatures and features adjustable pore size (C_3_H_2_BO; Figure 11).

Hyunchul et al.’s research was conducted from four aspects, namely the synthesis of COFs, material characterization, gas adsorption performance study, and thermal desorption spectroscopy (TDS) experiments [36]. Through these studies, researchers discovered that introducing pyridine molecules (Py) into the pore walls of COF-1 successfully reduced the pore size, which is crucial for improving the selectivity and adsorption capacity of the material. Furthermore, through the ABAB stacking mode, Py@COF-1 achieved tight packing between two layers, which helps to enhance the thermal and chemical stability of the material, while also providing more active sites for gas molecules, thereby improving adsorption efficiency. These findings provide a solid structural foundation for the application of Py@COF-1 in HIS.

In the research of gas adsorption performance, Py@COF-1 has demonstrated outstanding capabilities. Through precisely controlled experiments, researchers tested Py@COF-1’s adsorption capacity for hydrogen isotope mixtures at various temperatures. The experimental results revealed that within the low-temperature range of 19.5 to 70 K, Py@COF-1 not only exhibited excellent adsorption capacity for H_2_ and deuterium gas (D_2_) but also demonstrated unique adsorption–desorption behavior, indicating that the pore structure in the material exhibits flexibility with temperature variation. This low-temperature flexibility may be attributed to the presence of pyridine molecules in the pore walls of Py@COF-1, which may rearrange at different temperatures, thereby altering the pore size and affecting the adsorption and desorption of gas molecules. This temperature-responsive pore size variation provides a novel mechanism for the separation of hydrogen isotopes, making Py@COF-1 potentially valuable for applications in HIS.

In the thermal desorption spectroscopy (TDS) experimental section, researchers utilized a self-built TDS device to conduct adsorption tests on Py@COF-1 with a mixture of H_2_ and D_2_ gases. The aim of this experiment was to directly assess the adsorption selectivity of Py@COF-1 towards the two hydrogen isotope molecules under specific conditions. The experimental results revealed that the adsorption capacity of Py@COF-1 for D_2_ is significantly higher than that for H_2_, indicating that the material can effectively distinguish and preferentially adsorb heavier isotope molecules. This selectivity may stem from the stronger interaction between D_2_ molecules and Py@COF-1, as well as differences in diffusion kinetics of D_2_ molecules within the pores of Py@COF-1. Additionally, the experiment observed the characteristic variation in adsorption–desorption behavior of Py@COF-1 with temperature, further confirming the low-temperature flexibility of the material’s pore size.

These findings not only confirm the potential of Py@COF-1 in HIS but also provide experimental evidence for its practical application. Specifically, Py@COF-1’s highly selective adsorption of D_2_ under low temperature conditions positions it as a strong candidate material for HIS in applications like nuclear fusion. In terms of industrial application potential, Py@COF-1’s high selectivity and low-temperature flexibility make it a strong candidate material for HIS in industrial applications, especially in scenarios where efficient separation of hydrogen isotopes is required, such as nuclear fusion reactions. Researchers anticipate that this study will inspire further research on pore size engineering of COFs and promote the development of more intelligent porous materials suitable for various isotope separations, including 3He/4He separation.

According to the research conducted by Hyunch al., we can delve into the durability of materials over time from several perspectives—firstly, thermal stability, which was examined through parametric analysis. Py@COF-1 experiences weight loss at 50 °C, corresponding to the loss of one Py molecule per boroxine ring. This suggests that Py@COF-1 exhibits excellent thermal stability below 150 °C, a crucial factor for ensuring long-term stability in practical applications. Furthermore, from the perspective of structural stability, scanning electron microscopy (SEM) data demonstrate that Py@COF-1 exhibits a sheet-like morphology akin to its parent COF-1, indicating that the material’s morphology is preserved during the synthesis process, a positive indication for structural stability. Additionally, the article mentions that by completely stripping the pyridine (Py) molecules from Py@COF-1, clean-1 can be obtained, underscoring the material’s ability to maintain structural integrity during chemical treatment.

In terms of chemical stability, infrared spectroscopy (IR) and nuclear magnetic resonance (NMR) spectroscopy were employed for analysis, revealing a strong correlation between the spectra of Py@COF-1 and the model compound (Ph_3_B_3_O_3_·Py). This suggests that the material retains its structural integrity and composition within a chemical environment. Furthermore, the article delves into the durability aspect, highlighting the unique adsorption behavior of Py@COF-1 at low temperatures ranging from 19.5 to 70 K. Notably, the hysteresis phenomenon observed at 30 K underscores the material’s dynamic pore size modulation capability at low temperatures. This inherent “flexibility” is likely to positively impact the material’s long-term durability, as it enables adaptive pore size adjustments under varying temperature and pressure conditions. In conclusion, Py@COF-1 exhibits favorable characteristics in terms of thermal stability, structural stability, chemical stability, and durability, all of which contribute to its enhanced long-term stability and durability.

POC stands for “Porous Organic Cage”, representing a special type of organic compound formed by covalently linking organic monomers to create highly symmetrical cage-like structures. These molecules exhibit the following characteristics: POCs possess highly ordered porous structures with cavities and channels, permitting the entry and passage of small molecules or ions. They consist of lightweight organic materials, including carbon, hydrogen, oxygen, nitrogen, and other elements, forming a stable cage-like framework. The pore size and internal functional groups of POCs can be tailored through chemical synthesis methods to cater to various application needs, such as gas storage, separation, and catalysis. Owing to their porous structure, POCs typically exhibit a high specific surface area, enhancing gas adsorption and storage capabilities. Many POCs adopt crystalline structures in the solid state, providing high consistency and predictability in both structure and function. POCs have demonstrated potential application value in fields such as gas separation, molecular recognition, drug delivery, and energy storage.

**Figure 12:**
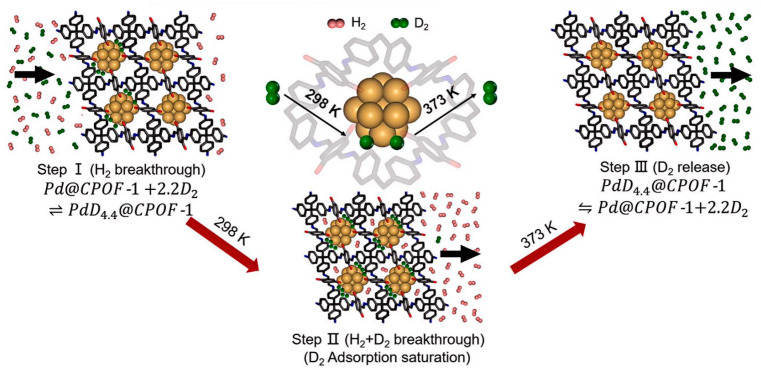
HIS mechanism of Pd@CPOF-1. At 298 K, the Pd nanoparticles selectively react with D_2_ to form the Pd–D bond in the confined space between the Pd nanoparticles and the CPOF-1 skeleton. At 373 K, the Pd–D bond appears to break so that D_2_ is released from Pd@CPOF-1 [56].

Jingru Fu et al. modified the internal cavities of POCs using organic synthesis methods, successfully preparing hybrid materials with outstanding quantum sieving capabilities as shown in Figure 12 [56]. By precisely adjusting the internal cavities of organic cage-like molecules (POCs) through organic synthesis, they crafted a novel cocrystal material, Cocryst1, which combines high selectivity and adsorption capacity, demonstrating exceptional HIS performance. At 30 K, Cocryst1 exhibits a D_2_/H_2_ selectivity of up to 8.0 and a D_2_ adsorption capacity of 4.7 mmol/g. Its pore size demonstrates temperature dependence. Molecular dynamic simulations and quantum mechanic calculations validated the experimental findings, shedding light on separation mechanisms encompassing pore size regulation, temperature dependence, quantum effects, and molecular diffusion dynamics within the pores of POCs. These combined factors have facilitated the effective separation of hydrogen isotopes, paving the way for innovative strategies in designing future efficient separation materials.

Jingru Fu and Hyunchul et al. have focused on HIS technology, particularly utilizing the QS effect to achieve the separation of H_2_ and D_2_ [56]. They also discussed the application of the QS effect in HIS, especially under low-temperature conditions, by exploiting the relationship between the pore size of materials and the de Broglie wavelength of hydrogen molecules to facilitate separation. Additionally, they employed TDS to evaluate the separation performance of the materials. However, Jingru Fu discussed modifying the internal cavities of POCs through organic synthesis methods to produce hybrid materials, specifically by combining small- and large-pore cage-like molecules to optimize separation performance. Hyunchul, on the other hand, focused on reducing pore size by introducing Py into the pore walls of COF-1 to achieve quantum sieving. In terms of material synthesis strategy, Jingru Fu employed a protection–deprotection strategy to synthesize a series of internally functionalized porous organic cage-like molecules, systematically adjusting the size of the cage-like pores through this method. Hyunchul synthesized Py@COF-1 through a Lewis base-assisted condensation reaction of 1,4-phenyldiboronic acid (BDBA) and discussed its structure and performance. In terms of structure and performance, Jingru Fu emphasized adjusting the pore size by modifying the internal structure of POCs, without affecting the external cage-like structure or crystal stacking. Hyunchul provided a detailed description of the structure of Py@COF-1, including its layered stacking mode, and the low-temperature flexibility induced by pyridine molecules.

## 6. Conclusions

QS with higher selectivity than conventional methods could be developed as an effective alternative to HIS and address one of the keys to future commercial fusion energy. Key thermodynamic and material properties influencing the efficiency of quantum screening (QS) encompass pore size, temperature, pressure, chemical affinity (CAQS), kinetic quantum screening (KQS), material functionality, temperature responsiveness, flexibility, strong adsorption sites, and material stability and scalability. The pore size is pivotal to the KQS effect, as it dictates the diffusion behavior of molecules within nanopores. Variations in temperature and pressure, on the other hand, impact the kinetic and thermodynamic behaviors of molecules, subsequently affecting QS efficiency. Chemical affinity, particularly the open metal sites (OMSs) within MOFs, offers strong adsorption sites, enhancing the adsorption of heavier isotopes and consequently boosting separation efficiency. Material functionality, such as the presence of open metal sites, is crucial for achieving CAQS. The temperature responsiveness and flexibility of certain MOFs or PCPs enable pore size adjustments in response to temperature changes, thereby enhancing operational pressure. The existence of strong adsorption sites, like the open metal sites in MOFs, maintains high selectivity even at elevated temperatures. Ultimately, thermal, chemical, mechanical, and radiation stability are essential for QS efficiency in practical applications. Additionally, sustainable and scalable synthetic methods, long-term stability, and feasible regeneration and recycling capabilities are also critical attributes for new materials. Precise control over these parameters can enhance the efficiency of quantum screening, leading to more effective HIS.

Researchers have attempted to use a variety of porous materials to improve the efficiency of KQS (utilizing different diffusion rates) and CAQS (relying on differences in binding strengths associated with zero-point energies). In this review, we highlight and summarize the state of the art in the separation of hydrogen isotopes using porous frameworks such as MOFs or zeolites. We first explain the basic principles of KQS and CAQS separations. Then, the effect of pore size on KQS sieving is presented, and the optimal pore size for KQS is determined to be between 3.0 and 3.4 Å. After that, we introduce the strategy of local flexible pore size and gating effect to improve the efficiency of KQS. Next, CAQS is used as an alternative method for KQS-based adsorptive separation of hydrogen isotopes from microporous materials at ultra-low temperatures. The key to achieve higher separation factors is to increase the enthalpy of adsorption at the adsorption sites. The different chemical affinities of the isotopes at strong adsorption sites are exploited, and we present methods for designing and identifying strong binding sites. In conclusion, we believe that pore structures with high HIS factors should have these three characteristics: (1) suitable one-dimensional channel sizes to maximize the KQS, (2) open metal sites with high adsorption enthalpies, and (3) flexible narrow pores for gating effects or multiple QSs. Future design of effective porous frameworks to better combine KQS and CAQS features for the separation of hydrogen isotopes is clearly beneficial to maximize separation efficiency.

In the field of HIS, the research methods of MOFs and zeolite materials integrate experimental and theoretical calculations, including gas penetration experiments, adsorption measurements, theoretical calculations and simulations, consideration of quantum effects, structural design optimization, high-throughput screening, and application of performance evaluation indicators. The aim is to explore and improve the separation efficiency and selectivity of materials through a combination of experimental verification and theoretical prediction.

In the realm of HIS, the selection criteria for materials, pore size, and adsorption sites constitute pivotal technical parameters for achieving efficient separation. Both MOFs and zeolites have garnered extensive research attention due to their diverse structural configurations, high specific surface areas, and tunable pore characteristics. Specifically, maintaining a pore size within the range of 3.0 to 6.0 angstroms is imperative for kinetic quantum screening (KQS), whereas chemical affinity quantum screening (CAQS) heavily relies on robust adsorption sites present on the material surface, which are typically furnished by metal cations or unsaturated metal centers. These distinguishing features endow MOFs and zeolites with remarkable separation capabilities across a wide range of temperatures, from low to room temperature.

## Figures and Tables

**Table 1:** Summary of rigid and flexible MOF skeleton performance.

Materials	Diameter of Hole/Å	Pressure/Bar	Temperature/K	Selectivity
(Zn_3_(BDC)_3_ [Cu(Pyen)])	5.6 × 12.0	1	77.3	1.38
CuBOTf	2.0, 8.7	0.01	40/77	5.8/1.2
USTC-700	3.7 × 7.2	0.01	30	7.3
MFU-4	3.88/2.52	0.01	40/50	6.9/5.8
MIL-53(Al)	8.5 × 8.5	0.01	40	10.5
CoFa	4.0 × 5.0	1	25	44
Cu(I)-MFU-4/	9.1	0.01	90	7.1
CPO-27-Co	10	0.03	60	11.8
Cu(I)Cu(II)-BTC	6.2	1	25	37.9
MOF-74-IM	N.A.	0.01	77	26
UiO-67_Be	N.A.	N.A.	77	49.4
FMOFCu	3.6	0.01	25/70	14/4
Co_2_(m-dobdc)	9.8	0.01	77	4.3
Co(pyz) [Pd (CN)_4_]	4.0 × 3.9	N.A.	25	21.7
MOF-303	N.A.	1	25	21.6
DUT-8(Ni)	N.A.	0.8	23.3	11.6
Co-MOF-74	11	0.01	77	2
FJI-Y11	8.4	1	77	1.2
Ni_2_(olz)	22	1 × 10^−5^	77	5.6
Ni_2_Cl_2_BBTA	N.A.	0.01	77	4.5

**Table 2:** Summary table of HIS using KQS effect.

Compound	Pore Size (Å)	Uptake Amount (mmol/g)	Conditions	Selectivity (D_2_/H_2_)
Na-CHA	3.8	10.2	38 K/550 mbar	25.8
Ca-CHA	3.8	12.2	38 K/550 mbar	18.3
10X	9	N.A.	77 K/314 mbar	1.33
Y	6–7	N.A.	77 K/314 mbar	1.52
RHO	3.6	0.43	77 K/5 mbar	2.4
MS5A	5	0.78	77 K/5 mbar	2.6
MS13X	10	0.61	77 K/5 mbar	3.1
H-ZSM-11	5.5	N.A.	77 K/5 mbar	1.8
AVL	3.55	N.A.	25 K/1 mbar	190
BCT	2.55	N.A.	25 K/1 mbar	50,000
MVY	2.94	N.A.	25 K/1 mbar	26.9

**Table 3:** Summary table of HIS using CAQS effect.

Compound	H_2_ Adsorption Enthalpy (ΔH) kJ/mol	D_2_ Adsorption Enthalpy (ΔH) kJ/mol	Uptake Amount (mmol/g)	Conditions	Selectivity (D_2_/H_2_)
Cu(I)-ZSM-5	N.A.	N.A.	N.A.	100 K/10 mbar	24.9
Cu(I)-MOR	N.A.	N.A.	N.A.	120 K/10 mbar	18.5
AgY	−14.5	−12.7	1.3	77 K/10 mbar	9.12
Ag(I)-ZSM-5	−25.0	−22.5	0.09	70 K/10 mbar	9.1
MgX	−6	−3	N.A.	77 K/3.5 mbar	5.6
5A	−3.29	−2.30	8	30 K/50 mbar	8.8

## Data Availability

No new data were created or analyzed in this study.
